# Iatrogenic ureteral injury diagnosed after colon cancer surgery: A case report of a rare and challenging complication

**DOI:** 10.1016/j.ijscr.2024.110147

**Published:** 2024-08-10

**Authors:** Anis Hasnaoui, Racem Trigui, Ahmed Ghaieth Dhahak, Mariem Nouira, Mourad Gargouri, Imen Ganzoui

**Affiliations:** a Faculty of Medicine of Tunis, Tunis El Manar University, Department of General Surgery, Menzel Bourguiba hospital, Rue Djebal Lakhdar, 1006 Tunis, Tunisia; b Faculty of Medicine of Tunis, Tunis El Manar University, Department of radiology, Habib Bougatfa hospital, Rue Djebal Lakhdar, 1006 Tunis, Tunisia; c Faculty of Medicine of Tunis, Tunis El Manar University, Department of epidemiology, Rue Djebal Lakhdar, 1006 Tunis, Tunisia; d Faculty of Medicine of Tunis, Tunis El Manar University, Department of Urology, Habib Bougatfa hospital, Rue Djebal Lakhdar, 1006 Tunis, Tunisia.

**Keywords:** Iatrogenic ureteral injury, Colorectal surgery, Sigmoid neoplasms, Iatrogenic disease, Case report

## Abstract

**Introduction:**

Iatrogenic ureteral injury (IUI) is an unfortunate and rare complication during colorectal surgery. While IUI remains a rare event, short and long-term complications are life-threatening ranging from intraperitoneal urinoma to septic shock and a serious risk of permanent renal failure.

**Case presentation:**

An 88-year-old patient was admitted with symptoms of large bowel obstruction and underwent a laparotomy with a discharge colostomy. A week later, a second laparotomy was required for a non-functional retracted stoma, revealing a perforation in a sigmoid tumor. The patient then had an oncological sigmoidectomy with Hartman's colostomy. Postoperative findings indicated a left ureteral injury. Three weeks later, a ureterostomy was performed. Unfortunately, the patient succumbed to heart failure one week after the ureterostomy.

**Discussion:**

Low anterior and abdominoperineal resection of the rectum, along with sigmoid resection are the most frequent causes of ureteral injury in digestive surgery. The primary objective of management is to establish a continuous flow of urine to avert potential complications. Preventing IUI in colorectal surgery is of paramount importance. This process initiates in the preoperative phase with a meticulous assessment of ureteral and colic anatomy through comprehensive review of preoperative imaging.

**Conclusion:**

IUI remains a seldom-seen, and yet a very serious complication in colorectal surgery. It is imperative to prioritize both preoperative and intraoperative measures to prevent IUI, ensuring optimal outcomes. When the diagnosis of a IUI is established, a treatment strategy should be meticulously devised and executed by a skilled and experienced surgeon.

## Highlights

• Iatrogenic ureteral injury is a seldom-seen complication in colorectal surgery.
• It represents a devastating complication associated with significant morbidity and mortality rates.
• Preventing Iatrogenic ureteral injury in colorectal surgery is of paramount importance.
• Treatment can be intricate, requiring careful consideration of timing and the best technique for ureteral reconstruction.

## 1 Introduction

Iatrogenic ureteral injury (IUI) is an unfortunate and rare complication during colorectal surgery. While IUI remains a rare event, short and long-term complications are life-threatening ranging from intraperitoneal urinoma to septic shock and a serious risk of permanent renal failure [1,2]. Although treating ureteric injuries has seen a great evolution in the last decades, it remains a challenging entity even in contemporary practice. This work has been reported in line with the SCARE criteria [3].

## 2 Case presentation

An 88-year-old patient with a history of type 2 diabetes, hypertension, and laparoscopic cholecystectomy 10 years ago, was admitted to our surgery ward for symptoms of large bowel obstruction evolving for 24 h. An abdominal computed tomography (CT) scan showed a large bowel obstruction secondary to a metastatic sigmoid tumor. The patient underwent an urgent laparotomy, and a discharge colostomy was performed. At the end of the first week of the postoperative course, an abdominal examination revealed a non-functional retracted stoma associated with abdominal distension.

**Fig. 1:**
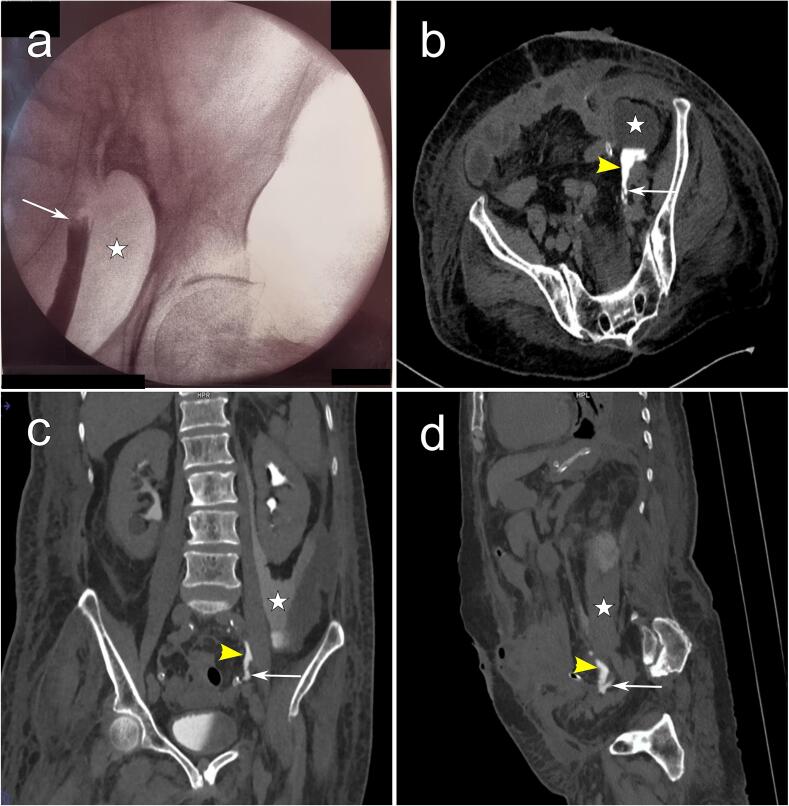
Imaging findings after the ureteral injury. (a) Retrograde ureterography showing a complete section of the left ureter (White arrow) with the presence of a urinoma (White star). (b) axial view, (c) coronal view and (d) sagittal view depicting the presence of an injury of the left ureter (White arrows) with extravasation of contrast agent (Yellow arrowheads) and a urinoma in the left iliac fossa (White stars). (For interpretation of the references to colour in this figure legend, the reader is referred to the web version of this article.)

The patient underwent a second laparotomy. Intraoperatively, a perforation in the sigmoid tumor without peritoneal effusion was identified. After a difficult dissection, the patient had an oncological sigmoidectomy with Hartman's colostomy. An active drainage was placed in the pouch of Douglas. Twenty-four hours later, urinary output in the urinary catheter was less than 100 cc, contrasting with intraperitoneal active drainage of 1600 cc of yellow-amber fluid. Further analysis of the drainage fluid was performed, showing creatinine and urea levels 10 times higher than normal values. Retrograde ureterography and CT urography showed an active extravasation of contrast on the left, associated with a urinoma extending from the left pararenal fossa to the left iliac fossa (Fig. 1). These findings suggested a left ureter injury. After a multidisciplinary meeting, we decided to perform a surgical nephrostomy. Percutaneous nephrostomy was not feasible as the urinary tract was not dilated.

**Fig. 2:**
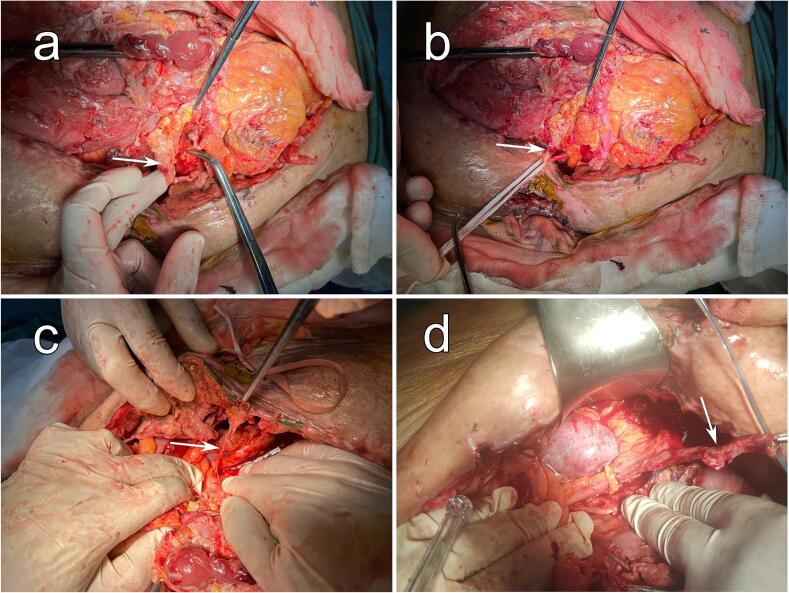
Intraoperative view. (a), (b), (c), and (d) sequentially illustrate the progressive stages of dissection, revealing the injured ureter (White arrows).

The patient was reoperated, three weeks later, after convenient preparation. Intraoperatively, a 300-cc urinoma located in the left iliac fossa was evacuated. We found a total section of the pelvic left ureter with necrosis of the distal segment (Fig. 2). After a meticulous and difficult dissection of the proximal segment of the left ureter, a ureterostomy was performed, along with a resection of the necrosed ureter's distal segment. Supplementary video provides more details of the intraoperative findings. One week later, the patient succumbed to the effects of heart failure.

## 3 Discussion

Low anterior and abdominoperineal resection of the rectum, along with sigmoid resection are the most frequent causes of ureteral injury in digestive surgery, with a documented overall incidence ranging between 0 and 1.5 % [1,4,5]. Identified risk factors of IUI encompass advanced cancer patients, malnourished patients, prior surgeries, obesity, history of radiation exposure, procedures in teaching centers, and operations related to endometriosis, diverticulitis, or bowel perforation [1,6,7]. In our case, the patient exhibited multiple risk factors, including advanced cancer, obesity, and a history of prior surgery. Ideally, a IUI is repaired directly if observed during surgery. Unfortunately, 50 to 70 % are still diagnosed in the postoperative phase [[8], [9], [10]]. When suspected, diagnosis is confirmed through retrograde ureterography and CT urography [11].

The primary objective of management is to establish a continuous flow of urine to avert potential complications. The majority of IUI cases manifest in the distal ureter. Given their proximity to the bladder, these injuries are typically addressed through ureteroneocystostomy. Dealing with complete sections of the abdominal ureter presents greater challenges, particularly if attempts at repair are made more than two weeks after the initial injury. In instances of delayed presentation (beyond two weeks) where ureteric stenting is not feasible, the conventional approach is to postpone surgery for a period of 3 to 6 months, allowing for the resolution of inflammation and tissue regeneration [12]. In our specific case, the injury was located in the pelvic ureter, and due to the associated uroperitoneum, the patient underwent a reoperation three weeks post-injury. We opted for a temporary proximal diversion, planning to reschedule the patient for auto-transplantation at a later date.

Preventing IUI in colorectal surgery is of paramount importance. This process initiates in the preoperative phase with a meticulous assessment of ureteral and colic anatomy through comprehensive review of preoperative imaging. Detecting any anomalies in urologic organs prior to surgery is crucial, as their discovery intraoperatively can pose a significant challenge for surgeons. Intraoperative precautions hinge on unequivocal identification of the ureter before proceeding with the ligation or section of the mesocolon. Moreover, in cases where anticipated ureter resection is warranted to adhere to carcinologic imperatives or whenever there is uncertainty regarding potential ureter injury, seeking assistance from a more seasoned and specialized surgeon is the right call [6]. Recently, the utilization of ureteral catheters prior to colorectal surgery has gained traction, although it remains a topic of ongoing discussion [9,13,14]. Studies have indicated that while catheter placement enhances the likelihood of intraoperative detection, it does not necessarily guarantee a reduced incidence of IUI. While further research is imperative, this tool may prove beneficial in intricate pelvic surgeries, cases involving advanced tumors, and in obese patients [6,7].

## 4 Conclusion

IUI remains a seldom-seen complication in colorectal surgery. It is imperative to prioritize both preoperative and intraoperative measures to prevent IUI, ensuring optimal outcomes. When the diagnosis of a IUI is established, a treatment strategy should be meticulously devised and executed by a skilled and experienced surgeon. Planning for treatment can be intricate, involving careful consideration of the opportune timing and the most suitable technique for ureteral reconstruction.

The following is the supplementary data related to this article.

Supplementary video Ureterostomy for Iatrogenic ureteral injury. Commencing with the initial stages of left ureter dissection, culminating in the creation of the ureterostomy by the conclusion of the intervention. Supplementary video

## Abbreviations

IUI: Iatrogenic ureteral injury
CT: Computed tomography

## Consent for publication

Written informed consent was obtained from the family for publication and any accompanying images after disidentification. A copy of the written consent is available for review by the Editor-in-Chief of this journal on request.

## Ethical approval

Ethical approval was deemed unnecessary by the institutional ethics committee of Menzel Bourguiba Hospital, Bizerta, Tunisia, as the paper is reporting a single case that emerged during normal practice. Ethical approval is not required in our institution for case reports.

## Funding

Nothing to declare.

## Author contribution

Anis Hasnaoui: Conceptualization, Writing-Reviewing and Editing. Racem Trigui: writing-Original draft preparation. Ahmed Ghaieth Dhahak: Data curation. Mariem Nouira: Data curation. Mourad Gargouri: Data interpretation. Imen Ganzoui: Data curation. All authors read and approved the final manuscript.

## Guarantor

Anis Hasnaoui

## Conflict of interest statement

Nothing to declare.
